# An RORγt Oral Inhibitor Modulates IL-17 Responses in Peripheral Blood and Intestinal Mucosa of Crohn's Disease Patients

**DOI:** 10.3389/fimmu.2018.02307

**Published:** 2018-10-22

**Authors:** Helena Bassolas-Molina, Ernest Raymond, Mark Labadia, Joseph Wahle, Elena Ferrer-Picón, Mark Panzenbeck, Jie Zheng, Christian Harcken, Robert Hughes, Michael Turner, Dustin Smith, Elisabeth Calderón-Gómez, Míriam Esteller, Anna Carrasco, Maria Esteve, Isabella Dotti, Ana Maria Corraliza, Maria Carme Masamunt, Clàudia Arajol, Jordi Guardiola, Elena Ricart, Gerald Nabozny, Azucena Salas

**Affiliations:** ^1^Department of Gastroenterology, IDIBAPS, Hospital Clínic, CIBERehd, Barcelona, Spain; ^2^Department of Immunology and Respiratory, Boehringer Ingelheim Pharmaceuticals Inc., Ridgefield, CT, United States; ^3^Department of Small Molecule Discovery Research, Boehringer Ingelheim Pharmaceuticals Inc., Ridgefield, CT, United States; ^4^Department of Gastroenterology, Hospital Universitari Mutua Terrassa, Barcelona, Spain; ^5^Centro de Investigación Biomédica en Red de Enfermedades Hepáticas y Digestivas (CIBERehd), Madrid, Spain; ^6^Department of Gastroenterology, Hospital Universitari de Bellvitge-IDIBELL, Barcelona, Spain

**Keywords:** Th17, commensal antigens, T-cell-transfer, RORγt inhibition, Crohn's disease

## Abstract

**Background and Aims:** Despite the negative results of blocking IL-17 in Crohn's disease (CD) patients, selective modulation of Th17-dependent responses warrants further study. Inhibition of retinoic acid-related orphan receptor gamma (RORγt), the master regulator of the Th17 signature, is currently being explored in inflammatory diseases. Our aim was to determine the effect of a novel oral RORγt antagonist (BI119) in human CD and on an experimental model of intestinal inflammation.

**Methods:** 51 CD patients and 11 healthy subjects were included. The effects of BI119 were tested on microbial-stimulated peripheral blood mononuclear cells (PBMCs), intestinal crypts and biopsies from CD patients. The ability of BI119 to prevent colitis *in vivo* was assessed in the CD4^+^CD45RB^high^ T cell transfer model.

**Results:** In bacterial antigen-stimulated PBMCs from CD patients, BI119 inhibits Th17-related genes and proteins, while upregulating Treg and preserving Th1 and Th2 signatures. Intestinal crypts cultured with supernatants from BI119-treated commensal-specific CD4^+^ T cells showed decreased expression of *CXCL1, CXCL8* and *CCL20*. BI119 significantly reduced *IL17* and *IL26* transcription in colonic and ileal CD biopsies and did not affect *IL22*. BI119 has a more profound effect in ileal CD with additional significant downregulation of *IL23R, CSF2, CXCL1, CXCL8*, and *S100A8*, and upregulation of *DEFA5*. BI119 significantly prevented development of clinical, macroscopic and molecular markers of colitis in the T-cell transfer model.

**Conclusions:** BI119 modulated CD-relevant Th17 signatures, including downregulation of *IL23R* while preserving mucosa-associated IL-22 responses, and abrogated experimental colitis. Our results provide support to the use of RORγt antagonists as a novel therapy to CD treatment.

## Introduction

Crohn's disease (CD) is a chronic remitting and relapsing inflammatory bowel disease (IBD) whose incidence is increasing worldwide. While management of active disease and its complications has dramatically improved thanks to the introduction of biologics, the lack of response to available therapy, intolerance and/or loss of initial response still leads to intractable disease in a significant percentage of patients. Thus, the quest for new therapies, especially those that can change the natural history of the disease, continues.

Among the mechanisms involved in disease pathophysiology, the production of IL-17 by immune cells was initially described in animal models of intestinal inflammation and later on in patients suffering from IBD (1–3). More recently, our group described an exacerbated Th17 response toward bacterially derived proteins (FlaX, A4-fla2 and YidX) in patients suffering from CD (4). These increased bacterial responses were identified within the peripheral CD4^+^ T cell compartment of CD patients compared to controls, and were characterized by the production of larger amounts of IL-17A and overexpression of a number of Th17 signature transcripts (i.e., *IL17F, IL26, RORC, CCR6, CCL20*, and *PTGER2*) while showing similar expression of classical Th1 genes. Evidence of Th17 axis involvement is also strongly supported by the fact that IL-23 (a cytokine involved in stabilization and further maturation of the IL-17 response) has also been critically involved in experimental models of intestinal inflammation (5, 6). More importantly, an anti-IL23/p19 monoclonal antibody (mAb) has shown promising efficacy in CD phase II studies (7) and is currently undergoing further development (8).

However, blocking IL-17 in CD patients unexpectedly resulted in negative effects (9–11), despite being efficacious in other immune-mediated diseases (12). IL-17 blockade in CD patients led in some cases to disease worsening, presumably due to severe infections, including mucocutaneous candidiasis. As a consequence, complete blockade of IL-17 using mAbs has been excluded from the IBD-armamentarium. Nevertheless, the contribution of IL-23 responding and potentially bacterial-driven pathogenic IL-17 producing cells to disease activity should not be yet dismissed. Alternative strategies to modulate pathogenic IL-17 responses, while preserving immune competence toward fungi and pathogenic bacteria in the gut, need to be designed and explored. In that sense, retinoic acid-related orphan receptor gamma (RORγt) inhibitors offer one promising strategy. RORγt is the master transcriptional factor that regulates the expression of the Th17 signature both in T cells (including αβ as well as γδ T cells abundant in the intra-epithelial compartment) and group 3 innate-like cells (ILC3) (13, 14).

Several small molecules targeting RORγt have been identified and tested in murine cells and models. These compounds not only suppress Th17 differentiation and IL-17 production, but also reduce the severity of experimental autoimmune diseases (15–18) including the IL10^−/−^ model of colitis (19).

In view of all this, we investigate herein the impact of a novel small molecule RORγt inhibitor (BI119) in controlling CD-associated immune responses. Given the diversity of RORγt expressing cells, and their varying roles and contributions to disease in peripheral and mucosal sites, we tested the effects of the compound both in microbial specific peripheral CD4^+^ responses and in inflamed ileum and colon from CD patients.

Finally, the effects of this novel oral compound were evaluated *in vivo* using the CD4^+^CD45RB^high^ T cell transfer colitis model. This mouse model recapitulates the aberrant CD4^+^ T cell response to commensal bacteria wherein transferred naive T cells become activated by gut bacteria in SCID recipient mice and mount a strong immune response resulting in similar pathology to that found in CD (20).

## Materials and methods

Additional information is provided in Supplementary Methods.

### Study subjects

Patients diagnosed with CD (*n* = 51) by endoscopic, histological and radiological criteria were recruited for the study for blood or biopsy collection. Healthy subjects (*n* = 6) without any known underlying acute or chronic pathological condition served as control blood donors. Epithelial crypts were obtained from surgical resection specimens from non-IBD individuals (*n* = 5) undergoing surgery for colorectal cancer; a segment of healthy mucosa was collected at least 10 cm from the margin of the affected area. Supplementary Tables S1, S2 show the clinical and demographic characteristics from non-IBD subjects and CD patients. This study was carried out in accordance with the recommendations of ethics committees at the Hospital Clínic de Barcelona, Hospital Mutua de Terrassa and Hospital Universitari de Bellvitge-IDIBELL with written informed consent from all subjects. All subjects gave written informed consent in accordance with the Declaration of Helsinki. The protocol was approved by ethics committees at the Hospital Clínic de Barcelona, Hospital Mutua de Terrassa and Hospital Universitari de Bellvitge-IDIBELL.

### Compound description

The RORγt inhibitor BI119 (Boehringer Ingelheim Pharmaceuticals Inc., Ridgefield, CT, USA) was discovered by screening a small-molecule compound library. BI119 strongly bound to the human RORγ ligand-binding domain (LBD) and was active in an RORγ LBD reporter assay (Kd for RORγ LBD– 65 nM; IC50 for RORγ LBD reporter assay 260 nM). The compound showed high selectivity toward RORγt as demonstrated by a lack of significant activity against RORα (IC50 > 10 μM) and RORβ (IC50 > than 6 μM).

### Antigen stimulation of human PBMCs

Peripheral blood mononuclear cells (PBMCs) were isolated from heparinized peripheral blood by Ficoll (Sigma-Aldrich, Madrid, Spain) gradient centrifugation. Cells were cultured in X-VIVO 15 medium (Bio Whittaker, Lonza, Belgium) supplemented with 2% inactivated AB human serum (Sigma-Aldrich) for 7 days. PBMCs were cultured with the microbial commensal proteins FrvX (Prometheus Laboratories Inc., San Diego, CA, USA) and YidX (exonBio, San Diego, CA, USA) at 2 μg/ml. An unstimulated condition was used as negative control. Heat-killed *Candida albicans (C. albicans)* was kindly provided by the Microbiology Department, Hospital Clínic-IDIBAPS, Barcelona, Spain and was used at 1 colony-forming unit (CFU): 1 PBMC as a positive control for IL-17 producing T cells. Recombinant interleukin (IL)-2 (20 UI/ml) (R&D systems, Minneapolis, MN, USA) was added to the culture on day 3. For RORγt blocking experiments, PBMCs were cultured in the presence of BI119 at 1 μM or dimethyl sulfoxide (DMSO) (vehicle control, 1:10,000). On the seventh day, supernatants were centrifuged and stored at −20°C until assayed. PBMCs were washed with cold PBS, re-suspended in 600 μL of buffer RLT (Qiagen, Hilden, Germany) and stored at −80°C until RNA extraction.

### Human intestinal crypt isolation and culture

Non-IBD intestinal epithelial crypts were isolated from intestinal tissue as previously described (21). For short-term crypt culture, 40 isolated crypts/25 μl Matrigel (BD Biosciences) were plated and cultured in either complete crypt culture medium or in medium containing supernatants from activated sorted antigen-specific CD4^+^ T cells (more detailed information is provided in Supplementary Materials and Methods). Antigen-specific T-cell supernatants were obtained from re-stimulating sorted cells with corresponding antigen (FrvX or YidX) treated with or without BI119. After overnight culture of crypts, RNA was extracted.

### Culture of human biopsies

Intestinal biopsies (4–6 per patient) were obtained from inflamed areas (defined by the presence of ulcers) of the colon or ileum from CD patients. Biopsies were washed twice in RPMI 1640 medium (Lonza, MD, USA) supplemented with 10% heat-inactivated fetal bovine serum (FBS) (Biosera, France), 100 U/ml penicillin, 100 U/ml streptomycin and 250 ng/ml amphotericin B (Lonza), 10 μg/ml gentamicin sulfate (Lonza) and 1.5 mM Hepes (Lonza). Whole biopsies were divided in two wells and cultured in the presence of BI119 at 1 μM or vehicle control (DMSO, 1:10,000) at 37°C in humidified atmosphere containing 5% CO_2_ incubator for 18 h. Total RNA was isolated and transcriptional analysis was performed.

### RNA isolation from human samples

Total RNA was isolated using RNeasy mini kit (Qiagen, Hilden, Germany) according to the manufacturer's instructions. Purity and integrity of the total RNA were assessed using the 2100 Bioanalyzer (Agilent, Germany) and quantified with a NanoDrop spectrophotometer (Nanodrop Technologies, DE, USA). Only samples with an RNA integrity number (RIN) greater than 7.0 were used.

### Quantitative real-time polymerase chain reaction (qPCR)

Total RNA (250 ng) was transcribed to complementary DNA using a reverse transcriptase (High Capacity cDNA Archive RT kit, Thermo Fisher Scientific, Waltham, MA, USA). Quantitative real-time PCR (qPCR) was performed in an ABI PRISM 7500 Fast RT-PCR System (Applied Biosystems) using predesigned TaqMan Assays (Applied Biosystems). ACTB was used as a reference gene and arbitrary units (AU) were calculated relative to ACTB.

### CD4^+^CD45RB^High^ T cell transfer colitis model

This study was carried out in accordance with the recommendations of Boehringer Ingelheim's Institutional Animal Care and Use Committee.

CB6F1 female mice served as cell donors and female CB.17 severe-combined immunodeficient (SCID) mice as recipients (Jackson Labs, Sacramento, CA). Mice were allowed a minimum of 2 weeks for acclimatization in specific pathogen-free conditions with 12-h light/dark cycle. All mice were used at 8–10 weeks of age with access to food and water provided *ad libitum*. CB6F1 mice were humanely sacrificed and spleens collected on ice. Following homogenization of spleens and red blood cells lysis, CD4^+^ cells were enriched from pooled spleen cells using a commercially available negative selection kit (Stemcell Technologies, Vancouver, BC) following the manufacturer's protocol. CD4^+^ enriched cells were stained with Alexa Flour 488 conjugated anti-CD4 clone RM4-5, Alexa Flour 647 conjugated anti-CD45RB clone C363-16A and PE conjugated anti-CD25 clone PC61 (Thermo Fisher Scientific). CD4^+^CD45RB^high^ cells were sorted on a FACS Aria II (BD Biocsience). CD4^+^CD45RB^low^ cells were collected separately. Groups of recipient CB.17 SCID mice were injected intraperitoneally with (5 × 10^5^) purified CD4^+^CD45RB^high^ donor lymphocytes in 200 μL PBS. A separate group of recipients was injected with the same quantity of non-colitogenic CD4^+^CD45RB^low^ lymphocytes as a control. A group of CB.17 SCID normal mice was also used as a control. All mice were weighed and observed weekly for clinical signs of illness including piloerection, hunched posture, decreased skin turgor and eye crusting. Diseased animals were humanely sacrificed for analysis at 4 weeks post-transfer or when body weight loss exceeded 20% of starting weight.

### Compound dosing on mice

BI119 was suspended in MC/tween solution (0.5% methylcellulose, 0.015% polysorbate 80) using a dounce homogenizer until a uniform suspension was formed. Recipient mice that received CD4^+^CD45RB^high^ cells were administered either MC/tween or 100 mg/kg BI119 by oral gavage immediately prior to cell transfer and then twice daily until study termination.

### Data analysis

Statistical analysis was performed using Bioconductor tools in R (V. 3.4.2). In experiments performed with human samples, differences between continuous variables were tested with nonparametric test (unpaired or paired Mann-Whitney-Wilcoxon test). Error bars show the mean and standard error of the mean (SEM). *P*-values were adjusted by false discovery rate (fdr) and were considered statistically significant when equal or less than 0.05. Regarding mice experiments, significance testing was performed by one-way ANOVA and Sidak's multiple comparisons test. For the total sum histopathology score, the Kruskal Wallis test and Dunn's multiple comparison test was performed.

## Results

### Expression of TH17-related genes is reduced by BI119

In order to evaluate the efficacy of a small molecule antagonist for RORγt, we stimulated human PBMCs to secrete measurable amounts of IL-17 in response to microbial antigens. Here we used the fungi *C.albicans* as a positive control because it is a strong inducer of Th17 responses. In addition, we used two different *Escherichia coli*-derived proteins: FrvX, which was selected based on reported increased sera reactivity in CD (22), and YidX, which we previously showed as capable of inducing exacerbated Th17 responses in CD patients (4). Supplementary Figure 1 shows production of IL-17, IFNγ, and IL-5 by stimulated PBMCs from both non-IBD donors (*n* = 6) and CD patients (*n* = 6) (Supplementary Table 1, Patient group 1). YidX and FrvX induced the production of significantly higher concentrations of IL-17, but not Th1 and Th2-related cytokines, in CD patients compared to non-IBD controls. In contrast, as expected, *C. albicans* induced similar increases in IL-17 levels in controls and CD patients compared to unstimulated conditions.

**Figure 1:**
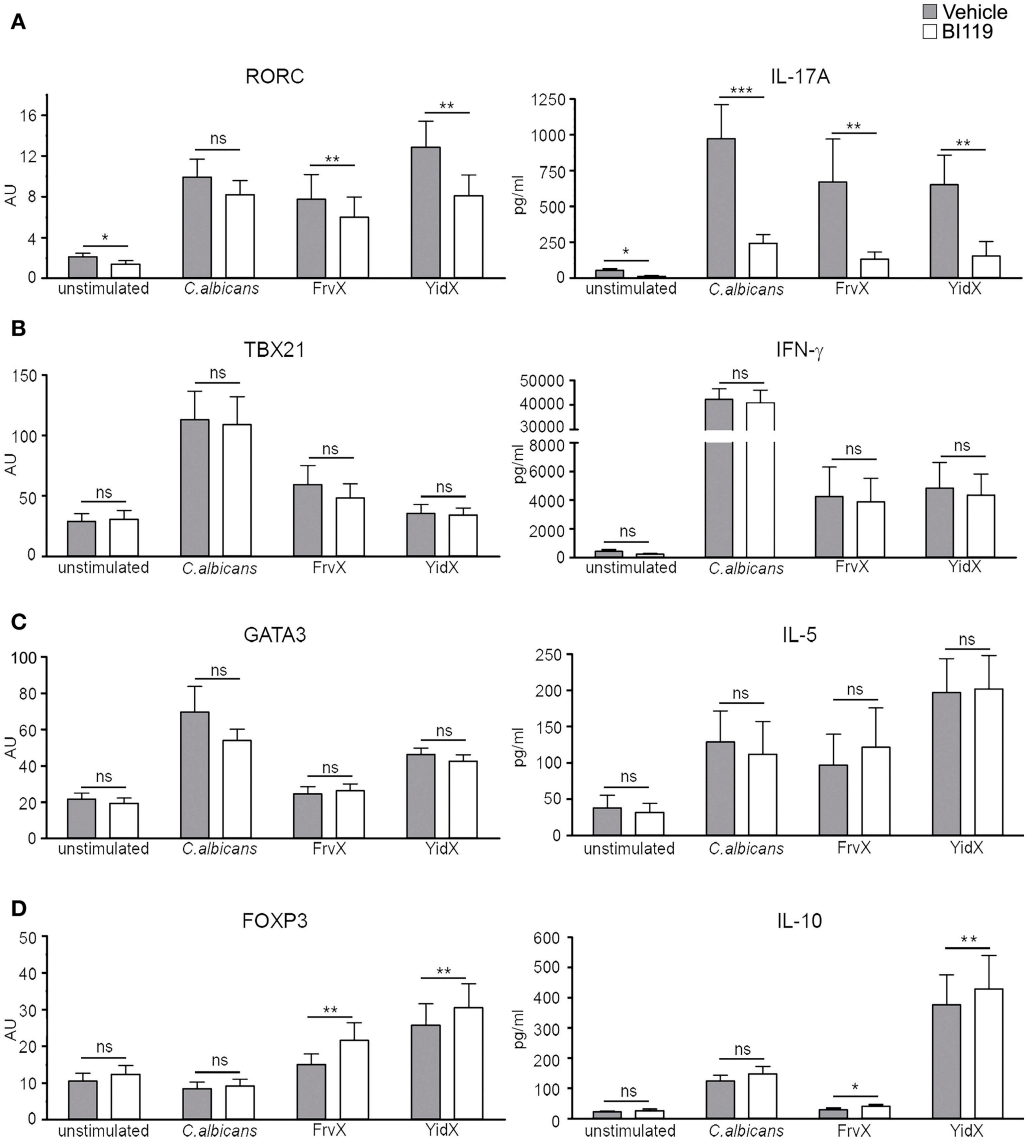
BI119 selectively inhibits Th17-related genes in PBMCs from CD patients. **(A)** Th17, **(B)** Th1, **(C)** Th2, and **(D)** Treg-related gene and protein expression were determined on PBMCs from CD patients (*n* = 12) after 7 days of culture in the presence of microbial antigens. Messenger RNA expression of transcription factors was assessed by real-time polymerase chain reaction. Protein production was measured by ELISA on PBMCs supernatants. Mean ± SEM. ns > 0.05, *P ≤ 0.05, **P < 0.005, ***P < 0.0005.

Using this system, we next tested the effect of a specific RORγt inhibitor (BI119) on the expression of a number of gene transcripts and proteins. PBMCs from an independent group of CD patients (*n* = 12; Supplementary Table 1, Patient group 2) were cultured alone or stimulated with *C.albicans*, FrvX, and YidX in the presence of BI119 (1 μM) or vehicle control. BI119 significantly reduced transcription of the Th17-related genes *RORC, IL17A, IL17F, IL22, IL26*, and *IL23R* under all conditions studied, while it did not alter *RORA* expression (Figure 1A and Supplementary Figure 2A). IL-17A (Figure 1A) and IL-22 (Supplementary Figure 2B) protein concentrations in culture supernatants from stimulated PBMCs were also significantly reduced by BI119 compared to vehicle-treated conditions. Remarkably, the Th1 or Th2 master regulators (*TBX21* and *GATA3* respectively), or their main effector cytokines (IFN-γ and IL-5), were not regulated by BI119 under any of the conditions studied (Figures 1B,C). In contrast, BI119 induced a small but significant (paired analysis, adjusted *p* < 0.05) increase in both *FOXP3* transcription and IL-10 production in microbial-stimulated PBMCs from CD patients (Figure 1D). These data demonstrate that BI119 strongly and specifically blocks Th17-related genes induced by microbial stimulation of CD PBMCs, though it does not interfere with Th1 and Th2 responses and up-regulates a Treg expression profile.

### BI119 reduces the pro-inflammatory effects of commensal antigen-specific CD4^+^ T cells on healthy human crypts

It has been described that IL-17 acts on epithelial cells by enhancing the expression of neutrophil and Th17 recruiting chemokines such as CXCL1, CXCL8 and CCL20, while it represses the Th1-attracting cytokine CXCL10 (4). Here we tested whether treating microbial activated CD4^+^ T cells with BI119 could reduce their inflammatory effect on the epithelial layer. We focused only on FrvX and YidX because their relevant responses in CD patients (4).

**Figure 2:**
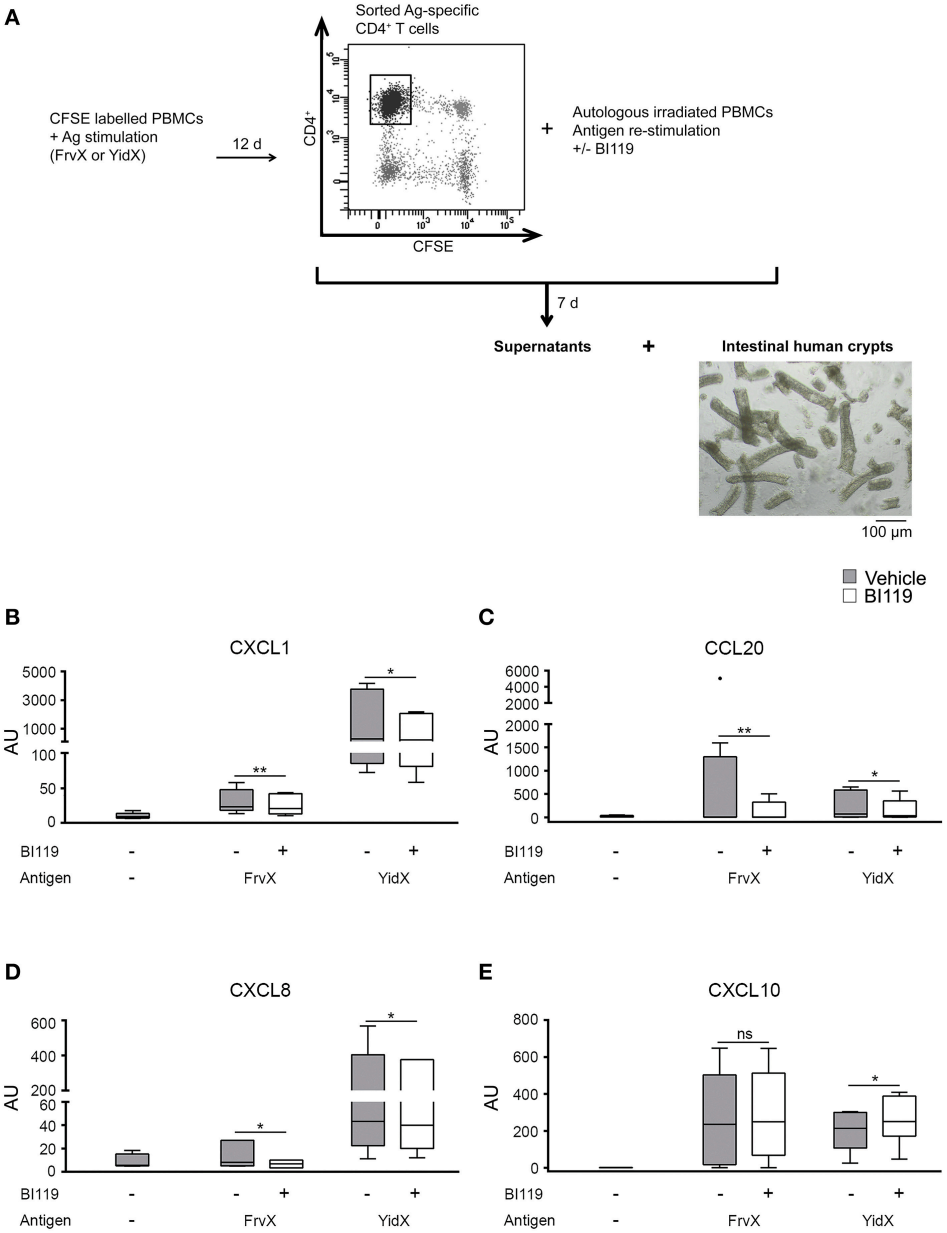
RORγt inhibition of activated antigen-specific CD4^+^ T cells reduces the expression of neutrophil-recruiting chemokines by intestinal epithelial crypts. **(A)** Schematic figure of experimental plan. CFSE-labeled PBMCs were stimulated with FrvX (*n* = 8) or YidX (*n* = 7) during 12 days. Responding CFSE^−^CD4^+^ T cells were sorted and re-stimulated with their cognate antigen and were treated or not with BI119. After 7 days supernatants were obtained from these cultures, and added to intestinal human crypts (*n* = 5). After overnight culture RNA of the crypts was extracted. Messenger RNA expression of **(B)*** CXCL1*, **(C)*** CCL20*, **(D)*** CXCL8*, and **(E)*** CXCL10* was assessed by real-time polymerase chain reaction. Mean ± SEM. ns > 0.05, *P ≤ 0.05, **P<*0.0*05.

In order to do this, CFSE-labeled PBMCs from CD patients (Supplementary Table 1, Patient group 3) were stimulated with FrvX (*n* = 8) or Yidx (*n* = 7). Responding CFSE^−^CD4^+^ T cells were sorted and re-stimulated with their cognate antigen in the presence of BI119 or vehicle control (Figure 2A). After 7 days supernatants from these cultures were collected and added to whole colonic crypts from non-IBD surgical specimens (*n* = 5). Supernatants from both FrvX and YidX-specific CD4^+^ T cells induced a marked up-regulation of *CXCL1, CXCL8, CCL20*, and *CXCL10* transcription on intestinal crypts (Figures 2B–E). Remarkably, supernatants from FrvX and YidX-specific CD4^+^ T cells treated with BI119 showed a significantly lower induction of *CXCL1, CXCL8*, and *CCL20* by intestinal crypts (Figures 2B–D). In contrast, *CXCL10* expression was either increased or unchanged under the same conditions (Figure 2E). Our results collectively indicate that the specific inhibition of RORγt on T cells modulates their pro-inflammatory effects on the epithelial lining.

### Effect of RORγt inhibition on inflamed intestinal mucosa from crohn's disease patients

Thus far we have shown that BI119 effectively inhibits RORγt-dependent gene transcription and protein secretion on circulating PBMCs responding to microbial antigen stimulation, specifically on CD4^+^ T cells. Expression of RORγt, however, is not limited to these lymphocyte populations. Indeed, intestinal mucosa cell types poorly represented in peripheral blood (i.e., intraepithelial lymphocytes and ILC3 cells) have been shown to express the Th17 transcriptional regulator.

Therefore, we investigated the effect of RORγt inhibition in intestinal tissue explants (endoscopic biopsies) obtained from the inflamed involved mucosa of CD patients (*n* = 18; Supplementary Table 2, Patient group 4) to assess the effect of BI119 on direct as well as indirect targets in tissue. Based on the differential cellular composition and transcriptional profiles between colonic and ileal mucosa, we analyzed separately the effects of RORγt inhibition on both intestinal locations. Samples were taken from inflamed colonic (*n* = 10) and ileal (*n* = 8) CD segments with comparable endoscopic disease severity based on their segmental CD endoscopic index of severity (CDEIS) score (Supplementary Table 2). Despite comparable disease severity, we found higher basal expression of *IL17A* and *S100A8* in ileal samples compared to colonic ones (Supplementary Figure 3). In contrast, expression of other genes regulated in inflammation as *CXCL1, CXCL8, IFNG, IL6* and *IL10* was comparable between colonic and ileal CD samples, suggesting that the differences in *IL17A* and *S100A8* expression may potentially reflect differences in disease location rather than degree of inflammation. The tissue specific marker *DEFA5* was, as expected, also significantly over-expressed in ileal compared to colonic CD.

**Figure 3:**
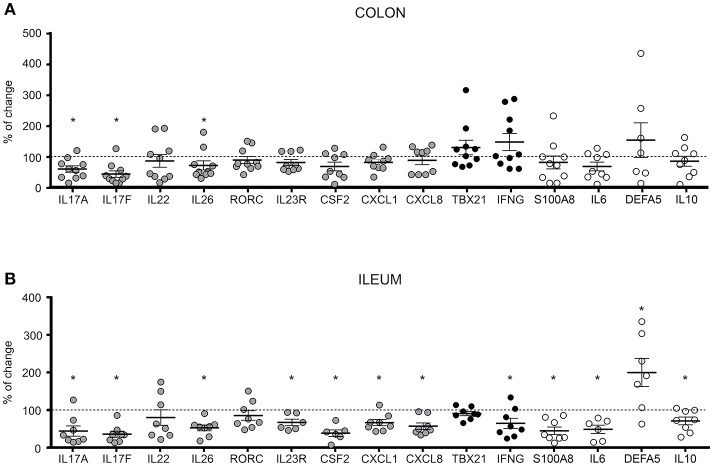
Differential expression of Th17, Th1 and CD-related genes in BI119- treated intestinal biopsies from active CD patients. Messenger RNA levels of untreated **(A)** colonic (*n* = 8–10) and **(B)** ileal (*n* = 6–8) biopsies were set as the 100% value for each donor (dashed line), and expression of BI119–treated samples was shown as the percentage thereof. Gray: Th17-related genes. Black: Th1-related genes. Mean ± SEM. ns > 0.05, *P ≤ 0.05.

Despite these variances in basal expression, treatment with BI119 significantly reduced transcription of *IL17A, IL17F*, and *IL26* in both the colon (Figure 3A) and ileum (Figure 3B) of CD patients with active inflammation. Remarkably *IL22* was not affected in either location, in striking difference to our results in PBMCs (Supplementary Figure 2). Other genes that are significantly regulated in CD mucosa compared to non-IBD controls (data not show) such as *IL23R, CSF2, CXCL1, CXCL8, IFNG, S100A8, IL6, IL10*, and *DEFA5* were also significantly changed by BI119 in ileal CD, whereas the effect on colonic biopsies did not reach statistical significance in most cases. These results suggest that BI119 can modulate some of the Th17-related genes, as well as inflammation-related genes, while preserving *IL22* expression in inflamed samples from active CD. Moreover, they indicate that ileal, compared to colonic, Th17 responses may be more amenable to modulation by RORγt antagonists.

In contrast to the experiments shown in Figure 1 using microbial antigen-expanded CD4^+^ T cells from PBMCs, BI119 did not significantly regulate the expression of *RORC* in tissue explants. Noticeably, the *RORC* gene can encode for 4 different isoforms, 2 of which give rise to the proteins RORγ and RORγt. The fact that RORγ can be expressed by most cells in the mucosa, including epithelial cells (data not shown), may explain the differences in *RORC* modulation between PBMCs and tissue explants. As the TaqMan assay used here does not differentiate between the two isoforms, we cannot accurately measure changes specifically related to RORγt using whole biopsy tissue.

### Treatment of T-cell-transfer colitic mice with BI119 ameliorates the disease

**Figure 4:**
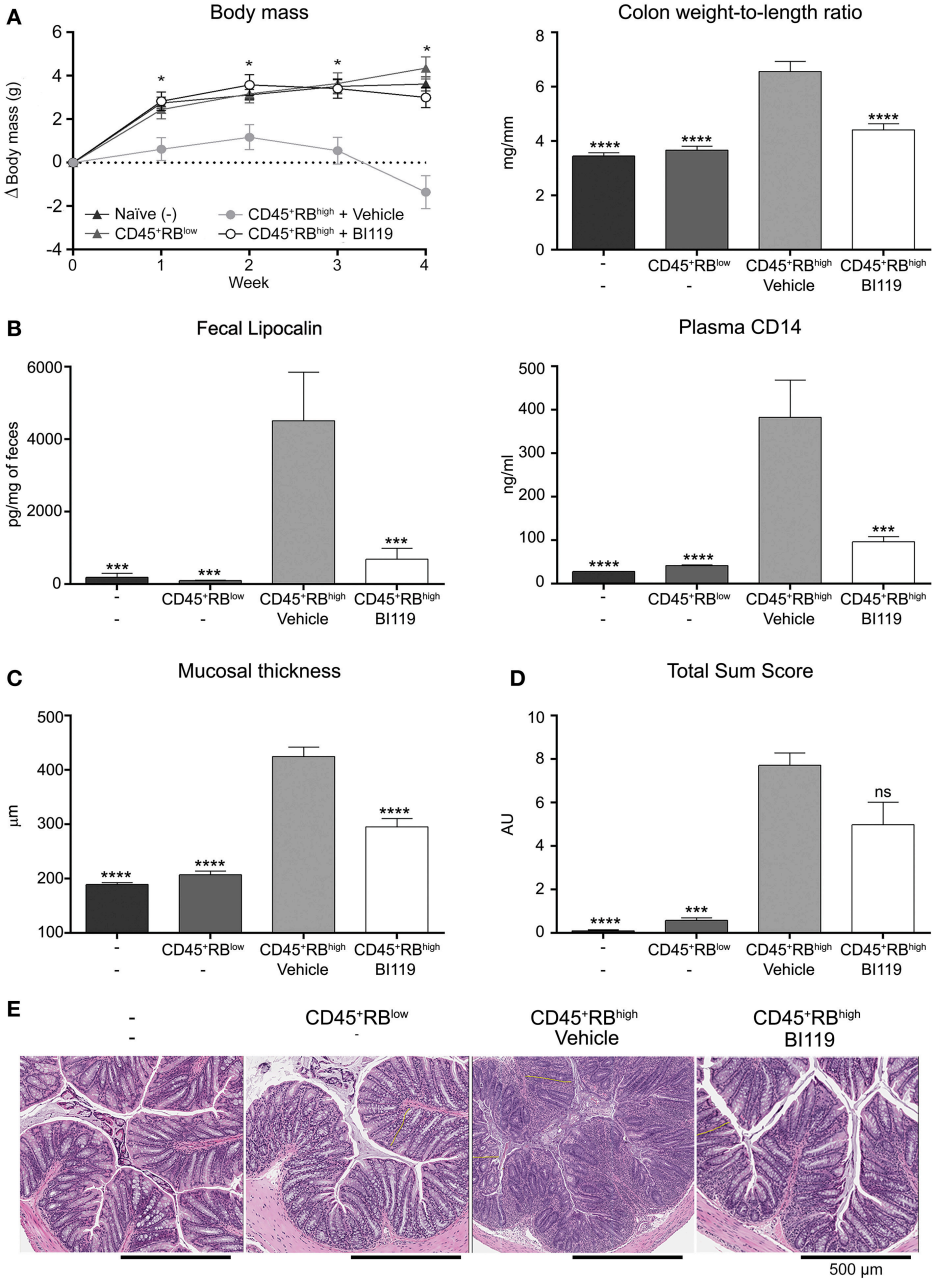
Impact of BI119 on T-cell-transfer colitic mice. **(A)** Body weight loss and colon tissue inflammation were reduced with BI119 treatment. **(B)** Fecal and systemic biomarkers were inhibited in BI119-treated colitic mice. Lipocalin was measured by ELISA on fecal supernatants. sCD14 was measured by ELISA on plasma samples. **(C)** Mucosal thickness was reduced in BI-treated colitic mice while **(D)** the histopathological score of colonic samples showed a trend but was not significantly decreased in BI119-treated colitic mice. **(E)** Hematoxylin and eosin stained histologic sections of the distal colon of different mice groups. Mean ± SEM. ns > 0.05, *P ≤ 0.05, ***P < 0.0005, ****P < 0.0001 compared to CD45^+^RB^high^ mice.

Finally, the efficacy of BI119 *in vivo* was assessed in a murine colitis model induced by transfer of CD4^+^CD45RB^high^ T cells into T- and B-cell-deficient CB.17 SCID mice. Mice that received CD4^+^CD45RB^high^ cells were orally administered either MC/tween (vehicle control) (*n* = 11) or 100 mg/kg BI119 (*n* = 12) twice a day for 28 days. Mice that were injected with a non-colitogenic CD4^+^CD45RB^low^ population (*n* = 9) and untreated CB.17 SCID mice (*n* = 9) were used as controls. Transfer of CD4^+^CD45RB^high^ T cells into immune-depleted mice induced a significant decrease in body weight, and a significant increase in macroscopic (colon weight-to-length ratio), histologic, fecal (lipocalin), and circulating (plasmatic sCD14) markers of inflammation at day 28 compared to both control groups (Figures 4A–E). Oral administration of BI119 significantly reduced both fecal lipocalin and circulating sCD14 compared to vehicle treatment (Figure 4B). A significant decrease in mucosal thickness due to a decrease in crypt hyperplasia was also observed in mice receiving BI119 (Figure 4C). Furthermore, administration of BI119 significantly reversed clinical and macroscopic signs of inflammation, while histologic improvement did not reach statistical significance (*p* = 0.18 vs. vehicle group) despite a clear amelioration on epithelial changes, mucosal inflammation and gland loss in compound-treated mice (Figures 4D,E and Supplementary Table 3).

**Figure 5:**
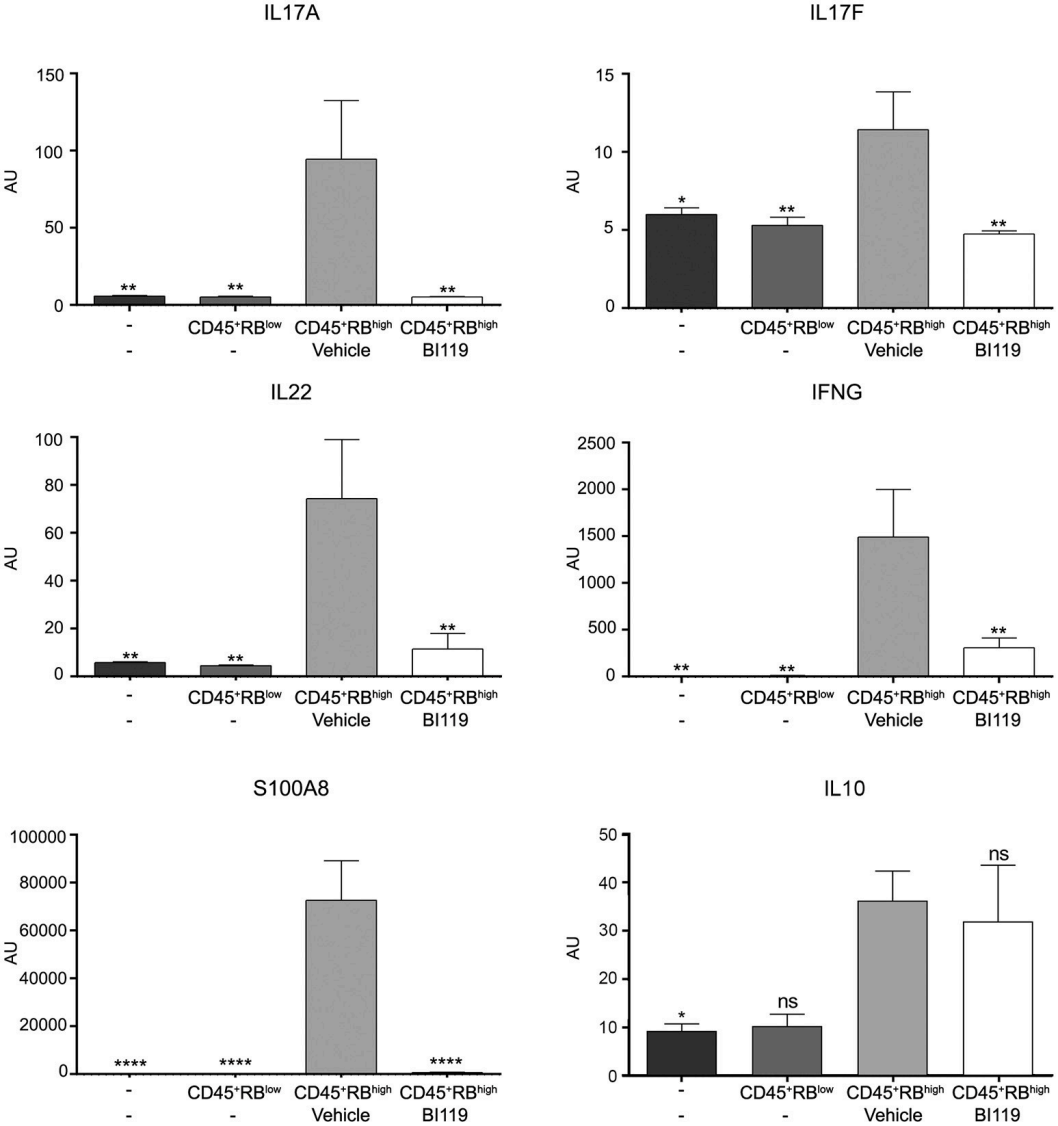
Gene expression changes in T-cell-transfer colitic mice after BI119 treatment. *IL17A, IL17F, IL22, IFNG* and *S100A8* expression was reduced in BI119-treated colitic mice. *IL10* expression was not altered using this treatment. Messenger RNA expression was assessed by real-time polymerase chain reaction. Mean ± SEM. ns > 0.05, *P ≤ 0.05, **P < 0.005, ****P < 0.0001 compared to CD45^+^RB^high^ mice.

We next looked at the molecular mechanisms involved in response to BI119 in this animal model by measuring the transcription of key inflammatory genes, including RORγt-dependent targets in the intestinal mucosa. As expected, colitis induced by CD4^+^CD45RB^high^ T cells in immune-depleted mice was associated with a significant increase in Th17 genes, *IFNG, IL10* as well as *S100A8* (Figure 5) and *S100A9* (data not shown). In agreement with the clinical, macroscopic and histologic protective effect of RORγt inhibition, oral BI119 treatment significantly reduced the expression of Th17-related genes such as *IL17A, IL17F*, and *IL22*, as well as *IFNG* and calprotectin (Figure 5). Remarkably, BI119 despite controlling all the other inflammation-related genes, did not significantly reduce transcription of *IL10* in the colon. Besides the influence on IL10, these *in vivo* experiments show a clear effect of oral BI119 administration in preventing T-cell driven murine experimental colitis.

## Discussion

We report here a novel small-molecule orally active inhibitor of RORγt as a potential strategy to treat CD. RORγt inhibition resulting from oral administration of VTP-43742, a small molecule antagonist that is known to be well tolerated and safe in healthy volunteers (23) and that has shown a signal of efficacy in a phase II study in patients with psoriasis (NCT02555709). In the context of IBD, evidence strongly supports the up-regulation and involvement of the IL-23/Th17 pathway in disease pathogenesis. However, results from clinical trials have shown that while blocking the IL-23 p19 subunit can be beneficial in CD (7), complete abrogation of IL-17 using mAbs has no benefit and even leads to disease worsening in some patients. Despite this undesirable effect, targeting of RORγt as an alternative approach to modulate pathogenic Th17 responses, while preserving protective ones, has been proposed. The key question remains how interfering with RORγt-dependent pathways will not result in the same undesirable effects observed upon IL-17 blockade in CD patients.

Two main cell lineages require expression of RORγt for their generation: Th17 (13), including a population of γδ intraepithelial lymphocytes (24), and the ILC3 subset comprising lymphoid tissue inducer (LTi)-derived cells (25) and natural cytotoxic receptor NKp46-expressing ILC3s. RORγt^+^ cells drive responses to extracellular bacteria and fungi by secreting several cytokines (IL-17A, IL-17F, IL-22, and IL-26) that promote neutrophil recruitment and epithelial microbial function (26). Remarkably, these cells are particularly abundant in the intestinal mucosa (27, 28) and have been described as being deregulated in intestinal inflammation (1–4, 29–31).

Both Th17 and ILC3s are characterized by their production of IL-17 and IL-22. Nonetheless, the requirement for RORγt beyond lineage determination appears to differ among these cell subsets, an important consideration when targeting RORγt with agonists or antagonists (16, 17). Data in mice models strongly suggests that while RORγt is necessary for lineage development of all RORγt-expressing cell populations, its expression acts predominantly to control IL-17 responses in the CD4^+^ compartment, while the ability to produce the Th17 signature by ILC3 occurs independently of RORγt function (19). This observation is further supported in human pediatric CD-isolated lamina propria T and ILC3s cells where GSK805 (an orally-available inhibitor of RORγt) inhibited *IL17* and *IL22* only on T cells but not on the lineage^−^ CD127^+^ lamina propria compartment (19). Therefore, while it is clear that ILC3s depend on RORγt for their development, it remains to be determined whether the nuclear receptor is also required for their maintenance and function.

All this evidence suggests that transient inhibition of RORγt activity effectively impacts T-cell-dependent Th17 responses in disease-relevant scenarios. In agreement with that, our results show that the effector Th17 function of CD peripheral blood bacterial (YidX and FrvX) responding cells can be significantly modulated by RORγt inhibition. Furthermore, we provide evidence that RORγt antagonism has a more profound effect on the transcriptional regulation of ileal CD compared to colonic inflammation. Given that Th17 are more predominant in the small intestine (32), where they are driven by specific microbial communities (33), it makes sense that the effects of RORγt would be more profound in this particular intestinal location. Finally, we show that RORγt inhibition significantly ameliorates T-cell-dependent intestinal inflammation in the CD4^+^CD45RB^high^ T-cell transfer experimental model, which supports the benefit of modulating Th17 responses *in vivo* to control intestinal inflammation. Other experiments conducted in this model demonstrated a more aggressive disease when transferred CD4^+^CD45RB^high^ T cells came from IL17A^−/−^ or IL17RA^−/−^ donor mice (34). In contrast, Leppkes et al. (35) and Krausgruber et al. (36) convincingly showed that adoptive transfer of Rorc^−/−^ CD4^+^ T cells into SCID recipients failed to induce colitis and this correlated with reduced IL-17A.

We should point out that at the doses used, BI119 treatment did not result in complete abrogation of IL-17 secretion by microbial-specific PBMCs, which would be a key difference with the effects exerted by a mAb used at saturating doses. This could represent a safety advantage in clinical use as it could potentially maintain responses to pathogenic bacteria and fungi.

Moreover, while BI119 significantly decreased production of IL-22 by PBMCs from CD disease patients, it did not regulate transcription of this cytokine in actively inflamed colonic or ileal biopsies. Besides antigen specific Th17 cells, IL-22 production is characteristic of ILC3s (37), a cellular type most abundant in the intestine and one that is significantly and selectively increased in CD compared with healthy donors or ulcerative colitis (UC) patients (31). These cells have been proposed to have a protective role in mucosal sites through their ability to produce IL-22, thereby inducing production of IL-10 by epithelial cells. IL-22 also protects epithelial cells from apoptosis through the activation of the transcription factor STAT3 (38, 39) and has regenerative properties on the epithelial stem cell compartment (39). Overall, preserving IL-22 production in the context of intestinal inflammation and homeostasis, despite RORγt inhibition, may offer an advantage. However, we should point out that the expression of *IL22* is known to be upregulated in the inflamed mucosa, both in CD and in experimental models of colitis (40). Indeed, mucosal healing leads to the control of *IL22* expression in colitic mice receiving BI119. This is not surprising, as one could expect that by protecting mice from developing colitis, BI119 would be acting not only against direct targets of RORγt (such as *IL17A*), but also on the expression of indirect targets (*IFNG* or *S100A8*) associated with the inflammatory response in colitis.

Besides the effects of BI119 on the signature of cytokine production by Th17 cells, we demonstrate that RORγt inhibition significantly downregulates transcription of the *IL23R* gene in response to microbial stimuli in peripheral blood and in the ileal mucosa of patients with CD, suggesting that one outcome of this pharmacological intervention is regulation of the response to IL-23 (a proven mediator and amplifier of disease in CD) (7).

An additional observed benefit of RORγt inhibition over IL-17 blockade is the potential activation of the Foxp3-dependent program upon RORγt antagonism. It is well known that the Th17 and the Treg differentiation programs compete with each other (41, 42). Indeed, RORγt physically associates with Foxp3 to antagonize each other's functions (15). We show here that BI119 specifically represses the Th17 signature while preserving the Th1 and Th2 pathways in PBMC cultures from CD patients stimulated with microbial proteins; in contrast, both *FOXP3* transcription and IL-10 production were upregulated in bacterial (YidX and FrvX) stimulated cultures. This observation is consistent with work from the Kuchroo's lab using murine Th17 cells differentiated *in vitro* (15). While neither our results nor previous studies (15) confirm the regulatory nature of the emerging T cells exposed to the RORγt antagonist, this observation suggests a potential added benefit of RORγt antagonism over IL-17 blockade. The possibility that RORγt antagonism could lead to a switch in the antigen-specific responses toward a tolerogenic phenotype in CD patients is highly intriguing and attractive; however, this remains only a hypothesis that would need in-depth *in vivo* validation. In agreement with this finding, mice receiving colitogenic CD4^+^CD45RB^hi^ T cells and the RORγt antagonist maintained higher *IL10* transcripts levels in tissue compared to non-colitic controls. While further studies are needed to understand the cellular source and mechanisms that result in this increased *IL10* expression in the non-inflamed colon, these results further strengthen our hypothesis that by interfering with RORγt we can promote regulatory functions. Overall, based on ours and previous results, we propose that RORγt inhibition can effectively modulate pathogenic CD-associated Th17 responses in periphery and mucosa while sparing innate sources of IL-17 and IL-22 at mucosal sites. This strategy would differ from blocking mAbs that non-selectively and completely block IL-17, which is required for control of extracellular bacteria and fungi. Furthermore, an intriguing possibility is that antagonizing RORγt, as opposed to IL-17 blockade, may lead to a transition of RORγt expressing cells into regulatory T cells, thereby resulting in a decreased sensitivity to IL-23 due to the downregulation of its receptor, a hypothesis that would need further exploration.

## Author contributions

HB-M designed and conducted experiments, acquired and analyzed data, and wrote the manuscript. ErR, ML, and JW designed and supervised experiments, MP designed and conducted experiments, acquired and analyzed data, JZ acquired and analyzed data, GN supervised experiments, RH, MT, CH, and DS identified and characterized antagonist. EF-P, EC-G, and ID designed and conducted experiments. MíE collected samples, and provided technical support. AMC performed biostatistics analysis. AC, MaE, MM, CA, JG, and ElR recruited patients and/or collected samples. AS designed the study, supervised experiments, analyzed data and wrote the manuscript.

### Conflict of interest statement

ErR, ML, JW, MP, JZ, CH, RH, MT, DS, and GN are Boehringer Ingelheim Pharmaceuticals Inc. employees. AS has received consultancy fees and grant money from Boehringer Ingelheim Pharmaceuticals Inc. The remaining authors declare that the research was conducted in the absence of any commercial or financial relationships that could be construed as a potential conflict of interest.

## Abbreviations

AU: arbitrary units
*C. albicans*: *Candida albicans*
CDEIS: CD endoscopic index of severity
CFSE: carboxyfluorescein succinimidyl ester
CFU: colony-forming unit
CRP: C-reactive protein
DMSO: dimethyl sulfoxide
EAE: autoimmune encephalomyelitis
ELISA: enzyme-linked immunosorbent assay
FACS: fluorescence-activated cell sorting
FBS: fetal bovine serum
fdr: false discovery rate
IL: interleukin-
ILC3: group 3 innate-like cells
LBD: ligand-binding domain
mAb: monoclonal antibody
mLN: mesenteric lymph nodes
PBMCs: peripheral blood mononuclear cells
PCR: Polymerase Chain Reaction
RIN: RNA integrity number
RORγt: retinoic acid-related orphan receptor gamma
SCID: severe combined immunodeficient.
