# Supplementary material for: An RORγt Oral Inhibitor Modulates IL-17 Responses in Peripheral Blood and Intestinal Mucosa of Crohn's Disease Patients
Source: Front Immunol. 2018 Oct 22;9:2307. doi: 10.3389/fimmu.2018.02307 (PMC6204372; doi:10.3389/fimmu.2018.02307)
Supplement: Supplementary file 1 [file Data_Sheet_1.docx]

Supplementary Material

**An RORɣt oral inhibitor modulates IL-17 responses in peripheral blood and intestinal mucosa of Crohn’s disease patients**

**Authors:** Bassolas-Molina H^1^, Raymond E^2^, Labadia M^2^, Wahle J^2^, Ferrer-Picón E^1^, Panzenbeck M^2^, Zheng J^2^, Harcken C^2^, Hughes R^3^, Turner M^3^, Smith D^3^, Calderón-Gómez E^1^, Esteller M^1^, Carrasco A^4,5^, Esteve M^4,5^, Dotti I^1^, Corraliza AM^1^, Masamunt MC^1^, Arajol C^6^, Guardiola J^6^, Ricart E^1^, Nabozny G^2^, Salas A^1^

*** Correspondence:** Azucena Salas. Department of Gastroenterology, IDIBAPS, Hospital Clínic, CIBERehd, Barcelona, Spain. Phone: +34-932272436. [**asalas1@clinic.ub.es**](mailto:asalas1@clinic.ub.es)

# Supplementary Data

**SUPPLEMENTARY MATERIALS AND METHODS**

**Measurement of soluble proteins**

Supernatants from cell cultures were harvested, centrifuged, and stored at -20ºC until they were assayed for soluble IFNγ, IL5, IL-22 and IL10 using commercially available enzyme-linked immunosorbent assays (ELISAs) Kits (R&D Systems). IL-17 was detected using purified and biotinylated antibodies and human IL-17 recombinant protein as a standard (Thermo Fisher Scientific).

**Cell sorting of human antigen-specific CD4^+^ T cells**

PBMCs were labeled with Carboxyfluorescein succinimidyl ester (CFSE) (Cell Trace CFSE cell proliferation kit, Thermo Fisher Scientific) at 5µM and cultured with FrvX and YidX proteins at 2µg/ml for 12 days. Recombinant-IL-2 (20 UI/ml) was added to the culture on day 7. Cells were harvested at day 12, washed and stained using a Live/Dead fixable violet dead cell stain kit (Thermo Fisher Scientific) and anti-CD4 PE mAb (RPA-T4; BD Biosciences, Franklin Lakes, NJ). Viable CFSE^-^ CD4^+^ cells were sorted in a Fluorescence-Activated Cell Sorting Aria II (BD Biosciences) and cultured with autologous irradiated PBMCs pulsed with 2 µg/ml of FrvX or YidX in the presence of BI119 (1µM) or vehicle control. IL-2 (20 UI/ml) was added on day 3. After 7 days of culture, supernatants were collected.

**Analysis of intestinal tissue from mice**

Colons were carefully excised; contents were removed before recording tissue weight and length. 2 cm of the middle colon was isolated and fixed in 10% formalin. An adjacent 1 cm piece was snap frozen in liquid nitrogen. Formalin-fixed paraffin-embedded colons were processed and stained with H&E according to standard procedures. Samples were scored by a pathologist blinded to the experimental treatments. Tissues sections (3-5 sections/mouse) were scored on a 0-5 scale based on 3 parameters: epithelial changes, mucosal inflammation and gland loss (Supplementary Table 3). Scores for each parameter were combined for a total sum score per mouse. In addition, mucosal thickness was obtained by measuring colon crypt length as an indicator of epithelial hyperplasia.

**Lipocalin measurement in fecal samples from mice**

Fecal samples in 1 ml of Tris buffer containing protease inhibitor (15-100 mg) were homogenized using a bead mill and centrifuged to remove insoluble matter. Supernatants were normalized by fecal weight to 10 mg/ml and assayed for lipocalin levels using a commercially available ELISA (R&D Systems) according to the manufacturer’s protocol. Data were converted to pg lipocalin per mg feces.

**sCD14 measurement in plasma samples from mice**

At sacrifice, blood was collected from mice by cardiac puncture into heparinized collection tubes to yield plasma. Plasma samples were assayed for mouse-soluble CD14 using a commercially available ELISA (R&D Systems) according to the manufacturer’s protocol.

**RNA expression analysis in mice samples**

Nucleic acid was isolated from frozen colon tissue samples by Trizol/chloroform extraction (Thermo Fisher Scientific) according to the manufacturer’s protocol. Samples were treated with DNAse I (Ambion Inc.) and purified RNA isolated using an Agencourt RNAdvance Tissue Kit with Biomek liquid handler (Beckman Coulter) according to the manufacturer’s protocol. RNA was reverse-transcribed using SuperScript VILO cDNA Synthesis Kit according to the manufacturer’s instruction. Gene expression levels were determined by real-time quantitative PCR using a ViiA 7 Real-Time PCR System using TaqMan™ Universal PCR Master Mix and TaqMan™ gene expression probes (Thermo Fisher Scientific) according to the manufacturer’s instructions. Data were normalized to 18S RNA and expressed as relative expression (2-ΔCT).

## Supplementary Figures


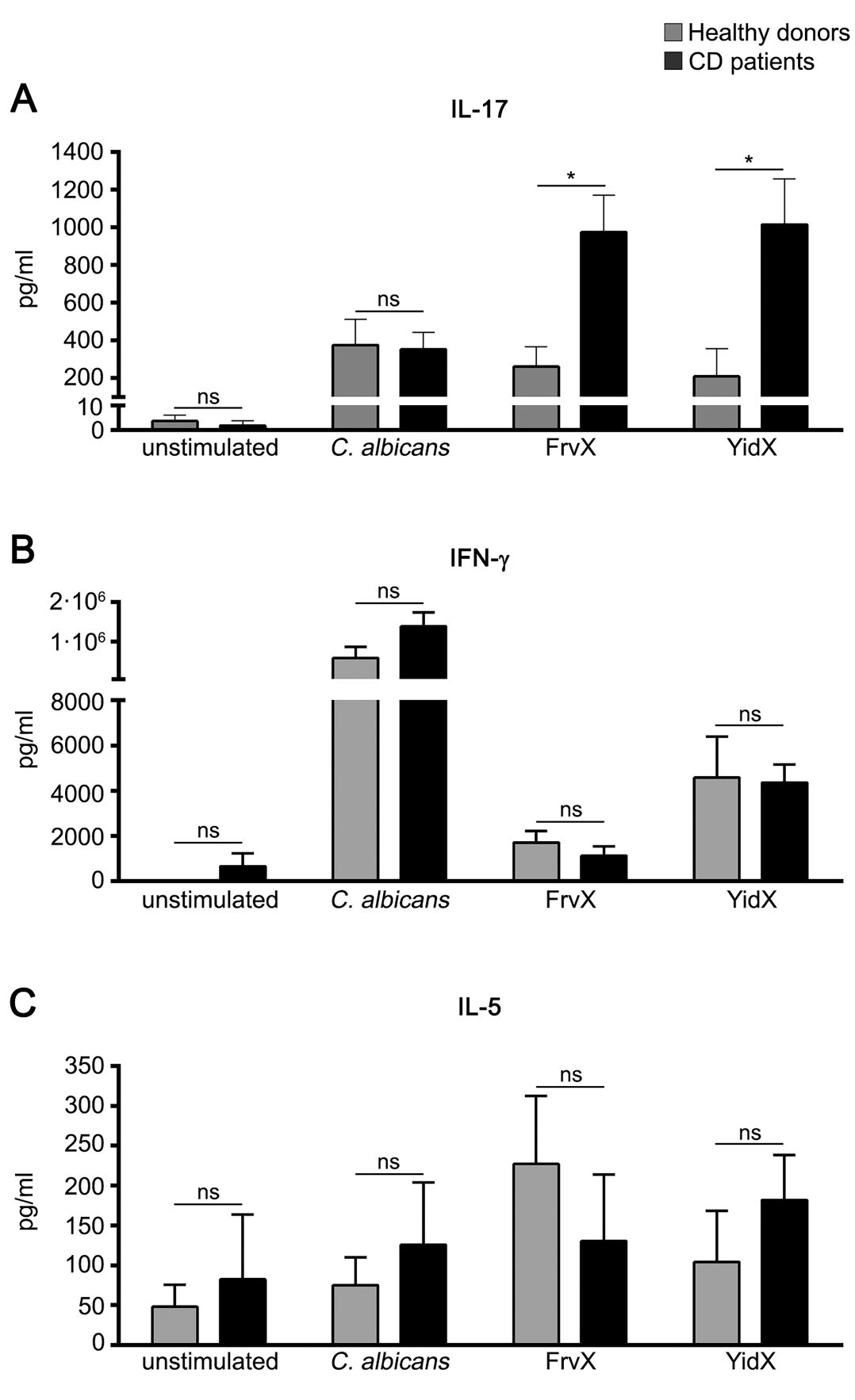


**Supplementary Figure 1. Higher IL-17 secretion by commensal antigen-stimulated PBMCs of CD patients.** (A) IL-17, (B) IFN-γ and (C) IL-5 were measured by ELISA in supernatants of PBMCs from healthy controls (n= 6) and CD patients (n=6) after 7 days of culture in the presence of microbial antigens. Mean±SEM. ns>.05, *P≤.05.

**

**

**Supplementary Figure 2. BI119 inhibits Th17-related genes in PBMCs from CD patients.** (A) Th17-related gene expression and (B) IL-22 secretion were determined on PBMCs from CD patients (n=12) after 7 days of culture in the presence of microbial antigens. Messenger RNA expression of transcription factors was assessed by real-time polymerase chain reaction. Mean±SEM. ns>.05, *P≤.05, **P<.005, ***P<.0005, ****P<0.0001.


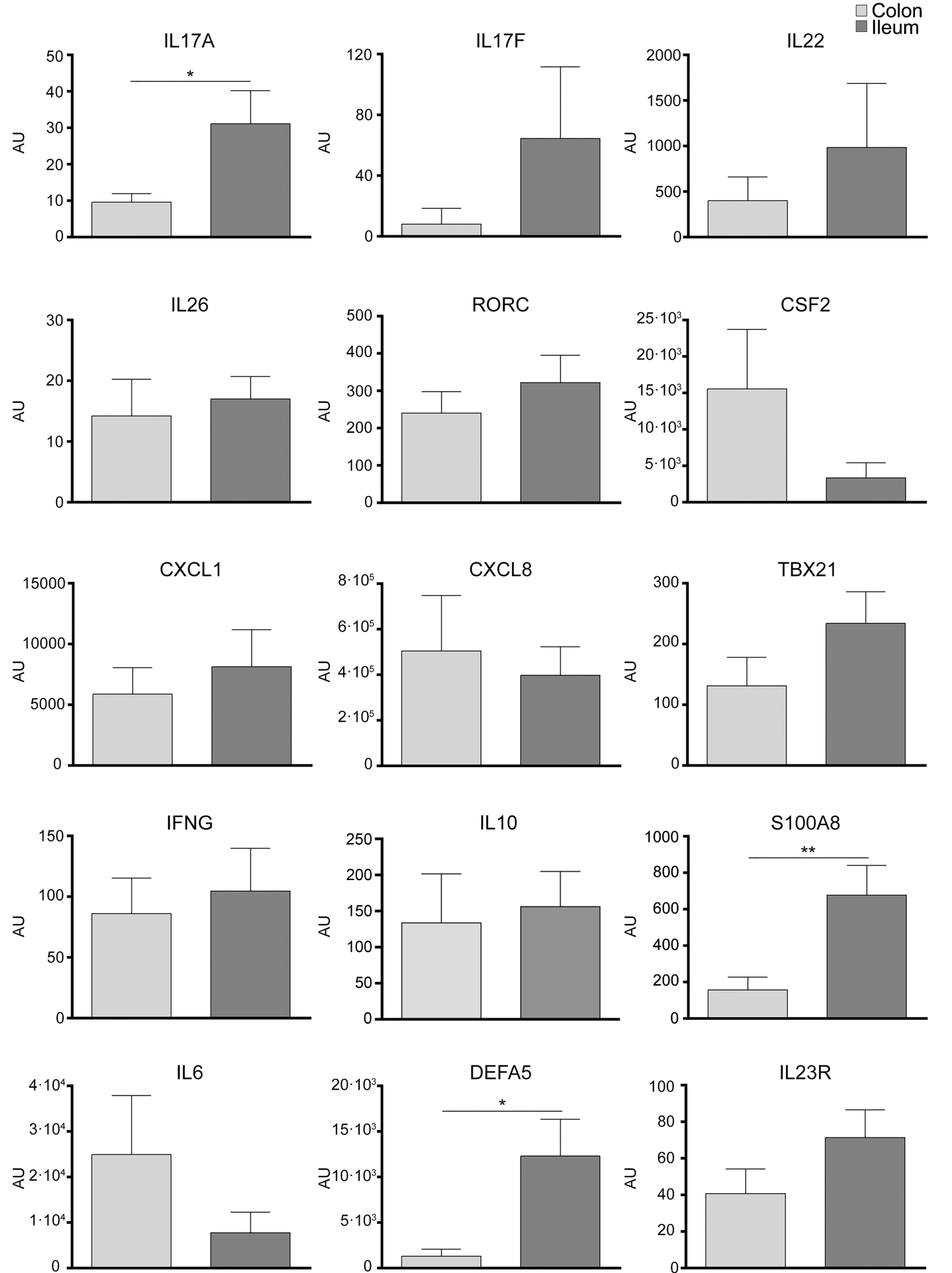


**Supplementary Figure 3. Differential gene expression between colonic and ileal inflamed biopsies from active CD patients.** Messenger RNA levels of colonic (n=8-10) and ileal (n=6-8) biopsies after 18 hours of culture. Mean±SEM. ns>.05, nominal P value *P≤.05., **P<.005.

|  | **N** | **Age** (years)  Median (range) | **Gender**  M/F | **Disease location** |  | **Treatment** | **Duration of disease** (years) Median (range) |
| --- | --- | --- | --- | --- | --- | --- | --- |
| Group 1 (Supplementary Figure 1) | | | | | | | |
| Controls | 6 | 37.3 (27-49) | 3/3 | - |  | - | - |
| CD patients | 6 | 47.8 (29-73) | 3/3 | 1/4/1/0 |  | 2/1/0/0/3 | 11.2 (1-24) |
| Group 2 (Figure 1 and Supplementary Figure 2) | | | | | | | |
| CD patients | 12 | 48.6 (28-74) | 7/5 | 4/3/5/0 |  | 3/2/0/0/7 | 11.7 (1-31) |
| Group 3 (Figure 2) | | | | | | | |
| CD patients  (stimulated with FrvX) | 8 | 46.9 (28-70) | 4/4 | 2/1/5/0 |  | 5/0/0/0/3 | 14.4 (6-42) |
| CD patients  (stimulated with YidX) | 7 | 39.4 (33-50) | 3/4 | 4/0/3/0 |  | 3/0/0/0/4 | 13 (8-18) |

**Supplementary Table 1. Description of the Crohn’s disease (CD) patients and controls.**

Gender: Male (M) and Female (F)

Disease location: L1 (ileal)/ L2 (colonic)/ L3 (ileocolonic)/ L2+L4 (colonic+upper disease)

Treatment: None/Mesalazine/Corticosteroid/Immunosuppressant/anti-TNF

|  | **N** | **Age** (years) Median (range) | **Gender**  M/F | **Disease location** | **Sample**  **location** | **Treatment** | **Duration of disease** (years)  Median (range) | **Global CDEIS** Median (range) | **Partial CDEIS** Median (range) | **Previous surgery**  Y/N | **CRP (mg/dl)**  Median (range) |
| --- | --- | --- | --- | --- | --- | --- | --- | --- | --- | --- | --- |
| Group 4 (Figure 3 and Supplementary Figure 3) | | | | | | | | | | | |
| Colonic samples | 10 | 37 (25-51) | 6/4 | 0/2/7/1 | 0/2/2/6/0 | 3/0/4/2/1 | 13 (0-28) | 18.16 (7.25-49.4)* | 18.5 (11.5-36) | 2/8 | 0.7 (0.06-7.60) |
| Ileal samples | 8 | 55 (25-73) | 5/3 | 3/0/5/0 | 8/0/0/0/0 | 3/1/1/3/0 | 3 (0-29) | 5.7 (3.4-19)* | 14 (3.4-38) | 6/2 | 0.342 (0.04-4.63) |

**Supplementary Table 2. Description of the Crohn’s disease (CD) patients that have been used in biopsy experiments.**

Gender: Male (M) and Female (F);

Disease location: Terminal ileum/Colon/Ileocolon/Upper gastrointestinal;

Sample location: Ileum/ Ascending colon/ Transverse colon/ Descending colon/ Rectum

Treatment: None/Mesalazine/Corticosteroid/Immunosuppressant/anti-TNF

Partial CDEIS of the area studied.

Previous surgery: Yes (Y) or not (N).* Mann-Whitney U test p.value = 0.01 (colonic samples vs ileal samples).

| **Parameters score** | **Epithelial cell hyperplasia, Goblet, Paneth cell depletion** | **Gland loss and erosions** | **Inflammation** | **Total SUM score** |
| --- | --- | --- | --- | --- |
| 0.5 | Very minimal | Very minimal, 1-2 focal areas of gland loss or mucosal erosion | Very minimal  1 or 2 small foci infiltration |  |
| 1 | Minimal 1-10%  Gland length 251-250um | Minimal 1-10% mucosa affected | Minimal 1-10% mucosa affected |  |
| 1.5-2 | Mild, 11-25%  351-450um | Mild, 11-25%  mucosa affected | Mild, 11-25%  mucosa affected |  |
| 2.5-3 | Moderate, 26-50%, 451-550um | Moderate, 26-50% mucosa affected | Moderate, 26-50% mucosa affected |  |
| 3.5-4 | Marked, 51-75%  551-650um | Marked, 51-75% mucosa affected | Marked, 51-75% mucosa affected |  |
| 4.5-5 | Severe, >76%,>650um | Severe, >76% mucosa affected | Severe, >76% mucosa affected |  |
| Average Score | Average of 4 cross sections | Average of 4 cross sections | Average of 4 cross sections | Combined parameters mean score |

**Supplementary Table 3. Total SUM score used for histologic analysis of murine colitis.**
